# Employment and return to work following chemoradiation in patient with HPV-related oropharyngeal cancer

**DOI:** 10.1186/s41199-016-0002-0

**Published:** 2016-06-03

**Authors:** Shrujal S. Baxi, Talya Salz, Han Xiao, Coral L. Atoria, Alan Ho, Stephanie Smith-Marrone, Eric J. Sherman, Nancy Y. Lee, Elena B. Elkin, David G. Pfister

**Affiliations:** 1 Head and Neck Oncology Service, Department of Medicine, Memorial Sloan Kettering Cancer Center, 300 East 66th Street, #1459, New York, NY 10065 USA; 2 Department of Medicine, Weil Medical College of Cornell University, New York, NY USA; 3 Epidemiology and Biostatistics, Memorial Sloan Kettering Cancer Center, New York, NY USA; 4 Department of Public Health, Weill Medical College of Cornell University, New York, NY USA; 5 Department of Radiation Oncology, Memorial Sloan Kettering Cancer Center, New York, NY USA

**Keywords:** Head and neck cancer, Employment, Survivorship, Quality of life, Chemoradiation

## Abstract

**Background:**

Human papillomavirus (HPV)-positive oropharyngeal cancer primarily affects working-age adults. Chemotherapy and radiation (CTRT) used to treat this disease may adversely impact a survivors’ ability to work after treatment.

**Methods:**

We surveyed participants with HPV-positive oropharyngeal cancer who completed CTRT regarding employment. We examined the associations between 1) sociodemographic and clinical factors and employment outcomes, and 2) health-related quality of life and satisfaction with ability to work.

**Results:**

102 participants were employed full-time at diagnosis for pay and surveyed at a median of 23 months post-CTRT (range 12–57 months). The median age at diagnosis was 57 years (range 25–76 years). During CTRT, 8 % stopped working permanently, 89 % took time off or reduced responsibility but later returned, and 3 % reported no change. For those who took time off but returned, median time to return to work was 14.5 weeks. In multivariable analysis, younger age predicted for needing more than the median time off. At time of survey, 85 % participants were working, 7 % had retired, and 8 % were not working for other reasons. Seventeen percent of participants were not satisfied with their current ability to work, which was associated with poorer health-related quality of life and persistent treatment toxicities (*p* < 0.001).

**Conclusions:**

CTRT interrupts employment in the majority of working patients with HPV-positive oropharyngeal cancer but most return. However, treatment-related toxicities might lead to dissatisfaction with ability to work.

## Background

The incidence of oropharyngeal cancer, a type of head and neck cancer, is rising despite declining rates of all tobacco-related malignancies in the United States [1, 2]. The rise in rates of oropharyngeal cancer is explained by an increase in cancers associated with human papillomavirus (HPV) [3]. Nearly 80 % of patients diagnosed with HPV-positive oropharyngeal cancer are male, and these patients are more likely to be non-smokers, non-drinkers, and generally healthier than patients with non-HPV-positive head and neck cancer [4–9]. They are also younger, with a median age at diagnosis of 57 years, about 5 years younger than head and neck cancer unrelated to HPV [1, 4].

Despite the growing appreciation for the difference in biology and better observed disease control in patients with HPV-positive compared to HPV-negative head and neck tumors, the management of oropharyngeal cancers is currently the same for patients regardless of HPV-status. Oropharyngeal cancer presents at a locally advanced stage in more than 70 % of patients and is often treated with a multimodality approach employing a course of definitive concurrent chemotherapy and radiation (CTRT) completed in seven weeks [10, 11]. Cisplatin remains the most studied and most commonly used radiation-sensitizing chemotherapy for the management of head and neck cancer, and the majority of head and neck cancers are treated with intensity-modulated radiation therapy (IMRT) [12–14].

The acute toxicities resulting from CTRT for head and neck cancer can include mucositis, difficulty with swallowing, pain requiring narcotic medications, anorexia, fatigue, thick saliva, nausea and vomiting and substantial weight loss. Taken together these toxicities contribute to a decline in general functional capacity [15, 16]. Longitudinal studies have reported that patients with head and neck cancer undergoing CTRT therapy can experience a clinically significant decline in quality of life and functioning and that recovery is a slow process which continues up to 12 months post treatment completion [17, 18]. Paradoxically, these effects may be amplified in the generally younger and otherwise healthier oropharyngeal cancer patients with HPV-positive tumors. In a recent report of oropharyngeal cancer patients treated with CTRT, patients with HPV-positive tumors had better baseline health-related quality of life, but a more rapid decline during treatment compared to patients with HPV-negative tumors. Both groups, however, showed substantial recovery by 12 months post-treatment, suggesting that the acute impact of CTRT is experienced more significantly by the younger, healthier patients [19].

Poorer health-related quality of life in patients with HPV-positive tumors may be particularly relevant to employment. Prior reports suggest that compared to survivors of other solid tumors, patients treated for head and neck cancer have difficulty returning to work after cancer treatment [20–23]. Some explanations for poorer occupational outcomes are receipt of multimodality therapy, post-treatment impact on swallowing and speaking, and possible disfigurement resulting from treatment [24–26]. However, because we do not yet fully understand their long-term health outcomes, it is unclear if these findings apply to the emerging cohort of working-age HPV-positive survivors.

Given the acute functional deterioration experienced during and after CTRT, and the increasing incidence of this diagnosis in working-age adults, our objective was to assess work interruption and employment outcomes in HPV-positive oropharyngeal cancer patients employed at diagnosis. We hypothesized that younger age and the receipt of high-dose cisplatin would be associated with more work interruption in this population.

## Results

The study sample included 102 patients who had been diagnosed with HPV-positive oropharyngeal cancer at least a year prior, who were working full-time at diagnosis, and who completed a survey about employment and quality of life (Table 1). Ninety-four patients (92 %) provided a job description, and the majority of these participants (87 %) were employed in jobs most often conducted in an office setting (Table 2). Participants were at a median of 23 months from completion of treatment with CTRT (range 12–57 months) at the time of the survey, and 94 % were male. At time of diagnosis, the median age was 56 years (range 25–75 years) and 90 % had a Karnofsky performance status (KPS) score of 90 %. A majority (85 %) had stage IVA or IVB disease, and 49 % had tumors that arose in the base of the tongue. All patients received some type of radiation-sensitizing chemotherapy, and all patients were treated using IMRT. High-dose cisplatin (100 mg/m2 every 3 weeks) was the most commonly used regimen (66 % of participants).

**Table 1:** Characteristics of the Cohort

Characteristic	Number
Total	102
Months since diagnosis, median (range)	23 (14–60)
Age at diagnosis (years)	
< 50	22
50–59	56
≥ 60	24
Male	96
Marital status	
Married	82
Unmarried	20
Charlson comorbidity score at diagnosis^a^	
2	24
3	50
≥ 4	28
KPS functional status at diagnosis	
90 %	92
80 %	10
Stage	
III	15
IVA/IVB	87
Site	
Base of tongue	50
Tonsil	44
Oropharynx NOS	8
Treatment	
CTRT alone	90
Induction + CTRT	8
CTRT + surgery	4
Chemotherapy	
High dose cisplatin	67
Other chemotherapy	35

*Notes*

Percentages not provided given N close to 100

^a^Age-adjusted Charlson comorbidity score calculated including localized solid tumor

**Table 2:** Job category and work interruption during concurrent chemotherapy and radiation

Job category (example)	Number	Did not take time off	Reduced hours	Took time off, returned		Took time off, never returned
		*N* (%)	*N* (%)	*N* (%)	Median time off (weeks)	*N* (%)
Professional (attorney, psychologist)	53	2 (4)	9 (17)	38 (72)	15	4 (8)
Other officers & mgrs (plant manager, postal supervisor)	9	1 (11)	2 (22)	6 (67)	12	0 (0)
Executives or senior officers (hotel owner, bank executive)	8	0 (0)	2 (25)	6 (75)	14	0 (0)
Sales workers (real estate agent)	8	0 (0)	3 (38)	4 (50)	14	1 (12)
Craft worker (stage hand, iron worker)	6	0 (0)	1 (17)	4 (67)	6.5	1 (17)
Other	10	0 (0)	0 (0)	9 (90)	18	1 (10)
Not provided	8	0 (0)	1 (13)	6 (75)	9	1 (12)
Total	102	3 (3)	18 (18)	73 (71)	14.5	8 (8)

*Notes*

Row percentages add to 100 %

Jobs categorized using Unites States Equal Employment Opportunity Commission Job Classification Guide

Other includes job categories with <5 patients: administrative support workers (4), service workers (1), and technicians (3)

Based on *n* = 73 who provided information

### Employment

Three (3 %) participants never reduced their work hours or ever stopped working; 18 (18 %) were able to keep working in some capacity during treatment; 73 (72 %) stopped working but eventually returned; and 8 (8 %) stopped working and never returned. Among those who reduced their hours or took time off, the median time to return to full-time work was 14.5 weeks, with a range of 1 to 52 weeks (Table 2). Forty-four patients (43 %) either never returned to work or took more than the median 14.5 weeks off.

At the time of the survey, 87 (85 %) patients were employed, with 82 working full-time and 5 working part-time. Seven patients (7 %) had retired; their occupations were professionals (4), craft worker (1), an executive (1), and an administrative support worker included as “Other” in Table 2 (1). Eight patients (8 %) were not employed, and six of them had stopped working at time of treatment. The remaining two returned to work but then later stopped.

For the eight patients who did not return to work after treatment was completed, no sociodemographic (age, marital status), disease (stage or site), treatment (radiation fields or chemotherapy) were associated with this outcome in an unadjusted analysis. However, at the time of the survey, older patients and those with higher Charlson comorbidity scores at diagnosis were less likely to be working (*p* < 0.05). In multivariable analysis, younger age at diagnosis was associated with taking off more than the median 14.5 weeks (or not returning to work at all) after treatment completion (*p* < 0.04).

### Quality of life and satisfaction with ability to work

Overall, participants reported very high global quality of life at the time of the survey. Mean global self-rated health status on the EuroQol-5D Visual Analog Scale (EQ-5D VAS) was 86 (standard deviation 13). Just over half of the patients (55 %) reported no issues in the five domains addressed by the EQ-5D which includes mobility, self-care, usual activities, pain/discomfort and anxiety/depression. However, 30 % reported some level of pain, and 22 % reported some level of anxiety. There were fewer reports of difficulty with mobility (13 %), usual activities (12 %) and self-care (3 %).

Of 101 patients who responded to a question regarding satisfaction with ability to work at time of survey, 17 % reported dissatisfaction with their current ability to work. In relation to current employment status, 7 of 82 patients working full-time (9 %), 3 of 5 patients working part-time (60 %), 1 of 7 patients who retired (14 %), and 6 of 7 who were unemployed (86 %) reported dissatisfaction with their ability to work.

**Fig. 1:**
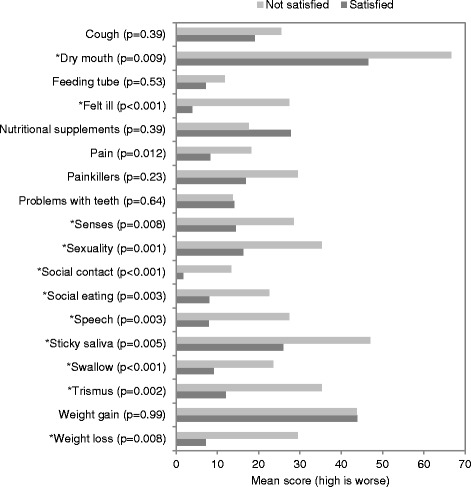
The European Organization for Research and Treatment of Cancer (EORTC) QLQ-H&N35 scale and single-item mean scores, by satisfaction with ability to work. *P* values shown are from the Kruskal Wallace exact test and those marked *are statistically significant at *P* ≤ 0.01

Participants who were dissatisfied with their ability to work reported more problems on the EQ-5D (*p* = 0.03) and lower mean EQ-5D VAS scores (70 versus 89, *p* < 0.0001) compared to those who were satisfied with their ability to work. Further, patients’ dissatisfaction with ability to work was associated with more severe late toxicities resulting from CTRT (Fig. 1). Specifically, participants who were dissatisfied with ability to work reported higher scores on the European Organization for Research and Treatment of Cancer Quality of Life Questionnaire Head and Neck 35 module (EORTC QLQ-H&N35) for weight loss, sticky saliva, dry mouth, trismus, swallowing, feeling ill, sexuality, social contact, social eating, speech and senses (*p* < .01).

When patients who were dissatisfied with their ability to work (*N* = 17) were asked about reasons for their dissatisfaction, most patients listed physical complaints or cognitive impairment. Fatigue was the most commonly listed reason (29 %) followed by memory loss and cognitive decline (18 %), chronic neck pain (12 %), difficulty speaking (12 %), unexplainable inability to work (12 %) and hearing loss (6 %). One patient reported that his employer did not approve his leave of absence for treatment.

## Discussion

This study highlights three important aspects of employment in survivors of HPV-positive oropharyngeal cancer treated with definitive CTRT. The first is that the majority of patients will be able to return to work following treatment and will be satisfied with their ability to work. The second is that younger age may be associated with longer employment interruption following CTRT. And the third conclusion is that symptoms related to treatment linger well past treatment completion and are associated with dissatisfaction with ability to work after treatment completion. Notably, type of chemotherapy, primary tumor location, baseline functional status, and sociodemographic variables were not associated with whether the participant returned to work.

Employment and return to work after cancer treatment are important issues for patients, not only because of the financial implications but also due to the self-identity and emotional well-being which can be associated with a job [23, 27]. Depending on the cancer population, the effects of cancer and its treatment on employment appear to be transient for the majority of patients, like the participants in our study. However, the impact is more permanent for a subset of survivors, which is consistent with our findings where 8 patients never returned to work after treatment completion [28, 29]. In a meta-analysis and meta-regression of 36 studies, including 20,366 patients with many different cancers and 157,603 age-matched controls, de Boer and colleagues estimated a pooled relative risk (RR) of unemployment of 1.37 compared to age-matched cancer-free controls from the general population [29]. Yet, there was a great deal of variability in employment status, treatments and toxicities associated with post-treatment unemployment across the studies included in the pooled analysis highlighting the heterogenous nature of cancer patients. Across cancer types, education and occupation appear to modify the effect of cancer on employment [20, 30]. For example, using the Finnish Cancer registry, Taskila-Abrant and colleagues reported that the rates of employment between cancer survivors 2–3 years after diagnosis was 9 % lower than matched cancer-free controls (RR 0.88, 95 % CI (0.86–0.90). However the difference was more pronounced in patients with only grade 1–9 education (RR 0.81, 95 % CI 0.78–0.84), and the difference was not significant in patients with university education (RR 0.96, 95 % CI 0.93–1.00) [31]. While we did not explicitly ask for educational level in our survey, the occupations represented in our participants are generally associated with higher education and our findings of high return to work following treatment completion are consistent with these prior observations.

In prior studies in the United States and Europe, patients treated for head and neck cancer were more likely to stop working and less likely to return to work after treatment compared to most other solid tumors [20, 22, 23, 25]. The association between job type and employment seen in other cancers is present in head and neck cancer survivors as well [26]. While we were unable to assess the association of job type on employment outcomes within our population, even in this study, where the majority of participants were employed in desk jobs, treatment was associated with some level of employment reduction, interruption, or discontinuation in all but 3 participants. Interestingly, younger age was associated with requiring longer employment interruption despite expected better physiologic tolerance to treatment than older patients. Although this study did not measure quality of life prospectively during treatment, others have reported that the acute toxicities of CTRT are more acutely experienced by younger patients with higher rates of depression and poor coping than older patients who might be physically less functional [32]. Our findings suggest that the toxicity of CTRT is significant in all head and neck cancer patients, regardless of the fact that patients with HPV-positive disease present healthier than their HPV-negative counterparts. However, despite needing time off, it is possible that a younger and healthier population is more likely to eventually recover and be able to return to work than historical head and neck cancer patients with HPV-negative tumors, explaining at least some of the differences between our results and prior studies on employment outcomes in the head and neck cancer population. Wells et al. recently reported that independent predictors of poorer quality of life in long-term head and neck cancer survivors include unemployment and younger age, but the relationship between the two was not elucidated in this cross-sectional survey either [33].

Only a few studies have evaluated both health-related quality of life and employment outcomes among head and neck cancer survivors. They reported that a higher severity of fatigue and oral dysfunction were associated with unemployment [24, 25, 34, 35]. In our study population, while most participants were able to return to work, the presence of long-term toxicities of CTRT, including fatigue, oral and social interaction symptoms, more than a year after treatment completion were associated with dissatisfaction with ability to work, most commonly reported by those unemployed at time of survey [24, 25, 35].

A limitation of this study is the high proportion of high-level managers and professionals in our single-institution study cohort. However, the epidemiology of HPV-positive oropharyngeal cancer is emerging as a disease of patients from higher socioeconomic status, and the employment demographics of this population have not been previously described [36, 37]. Another possible limitation is that the study population was predominantly male (94 %) which can further limit the generalizability of our findings. Marino and colleagues found that both higher educational level and male sex predict for a more rapid return to work suggesting a possible underestimation in our study cohort. [38] Further, 80 % of head and neck cancers are treated at cancer centers nationally, suggesting the tertiary-referral center may not bias the results toward higher socioeconomic status as much as in other cancer sites [39]. We did not ask for reasons for retirement; if some participants retired earlier than planned because of their health, this would increase the association between treatment toxicities and poor employment outcomes. In addition, while we asked about occupation, we did not explicitly ask about education which makes direct comparison against prior studies more difficult. Future studies should assess when participants began their time off from work and reasons for stopping work or retiring, which could further describe the trajectory of toxicity and subsequent recovery.

To our knowledge, this is the first study to look at employment outcomes specifically in patients with HPV-positive oropharynx cancer. Going into the survey, we expected to find that patients treated with cisplatin-based therapy would require more time to return to work given the higher toxicities generally attributed to this regimen. One possible explanation for our findings is that physicians are able to judge tolerability and each patient is pushed to their own extreme, and as a result, all are normalized in the end to very symptomatic, with no one regimen causing more harm in the end than another. Alternatively, it may be that CTRT, as a treatment, is simply toxic and requires time away from work and that differences between regimens are not detectable by a blunt instrument such as our retrospective analysis.

Unlike prior studies which have combined head and neck cancer disease sites and treatment, this study focused on a specific subset of patients who all underwent similar treatment with CTRT. Overall, the results of this study are reassuring in that the vast majority of patients are able to return to work compared to historical head and neck cancer patients. But even in this cohort, who generally is young and otherwise healthy at the time of diagnosis, there are areas for concern. Treatment toxicities, including dry mouth, sticky saliva, and weight changes, persist more than a year after treatment completion in many patients, and a subset of patients experiences prolonged employment interruption, an inability to return to work following CTRT, and dissatisfaction with ability to work. The results of this study will help patients and clinicians plan for the impact of cancer treatment on employment and highlight the on-going need for prevention, identification, and management of late toxicities.

## Conclusions

In this cross-sectional analysis of long-term survivors of HPV-related oropharyngeal cancer treated with CTRT, the majority of patients employed at baseline were able to return to work after taking a break from employment. However, almost all patients needed to take some time off from work (median 14 weeks) or reduce responsibilities during treatment, independent of baseline clinical or sociodemographic characteristics or treatment variables. There was a subset of patients who remained unsatisfied with their ability to work over a year from treatment completion, and this dissatisfaction was associated with worse functional and quality of life outcomes following CTRT therapy. Improved long-term toxicity management could therefore potentially enhance employment outcomes in this growing cohort of cancer survivors.

## Methods

We performed a cross-sectional survey regarding employment and quality of life among survivors of HPV-positive oropharyngeal cancer. The study was approved by Institutional Review Board at Memorial Sloan Kettering Cancer Center (MSKCC), and all study participants provided informed consent.

### Participants

We recruited patients seen in outpatient medical oncology or radiation oncology clinics at MSKCC from 2010 through 2014. Eligible patients had a pathologically confirmed oropharyngeal squamous cell carcinoma that was positive for HPV (either by HPV ISH or p16 on IHC in a CLIA approved laboratory), completed definitive CTRT at least 12 months prior and remained without clinical evidence of recurrence. The study team identified consecutive patients meeting study eligibility criteria before scheduled follow-up appointments with a medical oncologist. Of the 178 patients approached to participate, 5 patients refused or withdrew consent and 29 were found ineligible (never returned survey or HPV-status not confirmed). The sample was then limited to patients who reported full-time employment at the time of diagnosis (*n* = 102).

### Employment measures

The primary survey outcomes were related to employment at the completion of treatment and at the time of survey. Self-reported occupation at cancer diagnosis was categorized using the Unites States Equal Employment Opportunity Commission Job Classification Guide [40]. Participants were asked about employment status at completion of treatment, which was dichotomized as working (full-time or part-time) or not working (retired or unemployed). For those who returned to work, participants were asked to report whether they reduced their work hours, took time off from work, and if so, how much time was taken. We dichotomized time to return to work as longer than the median time for the cohort (or no return) versus shorter. Participants were asked about their current employment status, which was categorized as working or not working. We also asked participants about their satisfaction with their current ability to work, with response options of satisfied or not satisfied. In an open-ended item, participants could offer reasons for dissatisfaction with ability to work.

### Health-related quality of life measures

Health-related quality of life was estimated using the EQ-5D a validated instrument assessing level of impairment or function in five domains: mobility, self-care, usual activities, pain/discomfort and anxiety/depression. We dichotomized responses to these items as any problems or no problems [41]. The EQ-5D also includes a visual analogue scale (VAS) which asks participants is to rate their current health on a scale from 0 to 100.

The European Organization for Research and Treatment of Cancer Quality of Life Questionnaire Head and Neck 35 module (EORTC QLQ-H&N35) was used to assess head and neck cancer-specific quality of life. This instrument assesses pain, swallowing, senses, speech, social eating, social contact, sexuality, problems with teeth, dry mouth, sticky saliva, cough, trismus, weight loss, weight gain, use of nutritional supplements, feeding tubes, and painkillers [42]. The 35 items of the QLQ-H&N35 yield both multi-item symptom scale scores and single-item symptom scores, for a total of 18 distinct scores scaled from 0 to 100, with higher scores representing higher levels of symptomatology/problems [43].

### Covariates

Covariates included sociodemographic characteristics, disease and treatment characteristics, functional status, and comorbidity score at diagnosis. Clinical and demographic information (including age, sex, clinical stage, functional status at diagnosis, and treatment details) was collected from the electronic medical records. Functional status was measured by the Karnofsky Performance Score (KPS), ranging from 70–100 %, with higher scores representing better functional status. An age-adjusted modified Charlson comorbidity index score was calculated based on review of physician notes, with scores ranging from 1 to 6 [44].

### Statistical analysis

Patient characteristics and survey responses were analyzed using descriptive statistics, including frequencies, means and medians. Bivariate analyses, using chi-square and Fisher exact tests as appropriate, were performed between sociodemographic, disease, treatment, and clinical characteristics and the following outcomes: 1) no return to work, 2) longer time to return to work and 3) employment status at survey. We used multivariable logistic regression to evaluate whether sociodemographic, disease, treatment, and clinical characteristics (specifically age, marital status, stage, site, and chemotherapy) were associated with longer time to return to work (which included those who never returned to work). However, due to the small number of events, we did not use multivariable analysis to assess the relationship between sociodemographic, disease, treatment, and clinical characteristics and ever returning to work.

As a secondary employment outcome, chi-square tests were used to test the associations between satisfaction with ability to work at time of survey and each of the following predictors: EQ-5D global, self-care, usual activities, pain/discomfort and anxiety/depression responses. Because of the skewness observed in self-rated current health, the Mann–Whitney test was used to compare scores from the EQ-5D VAS between those who were and were not satisfied with their ability to work at the time of survey. To address concerns associated with multiple comparisons, we used a conservative p-value of ≤0.01 as the threshold for statistical significance in analysis of EORTC HN-35 scores [43]. All analyses were performed using SAS version 9.4 (SAS Institute, Cary, NC).
